# Formulation of new sourdough bread prototypes fortified with non-compliant chickpea and pea residues

**DOI:** 10.3389/fnut.2024.1351443

**Published:** 2024-06-06

**Authors:** Sara Cutroneo, Janos-Istvan Petrusan, Reiner Stolzenberger, Chiara Zurlini, Tullia Tedeschi

**Affiliations:** ^1^Food and Drug Department, University of Parma, Parma, Italy; ^2^Deutsches Institut für Lebensmitteltechnik e.V. (DIL), Quakenbrück, Germany; ^3^Bäckerei Reiner Stolzenberger, Bönnigheim, Germany; ^4^Stazione Sperimentale per l'Industria delle Conserve Alimentari, Parma, Italy

**Keywords:** legumes, agro-industrial residues, protein, bread, nutritional value

## Abstract

**Purpose:**

Nowadays, the promotion of a circular economy is fundamental to reduce food losses and waste. In this context, the possibility of using food supply chains non-compliant residues emerges. Much interest has been directed toward legume residues, in general and, in particular, to the possibility of combining different plant-matrices to improve nutritional profile, providing high-quality products.

**Methods:**

Five different formulations of breads, with a combination of seeds and cereals, were fortified with chickpea and pea protein concentrates. Samples were analyzed and compared with their relative control recipe to determine differences in composition, actual protein quality and integrity, and protein digestibility (performed with the INFOGEST method).

**Results:**

Samples showed a clear improvement in the nutritional profile with higher values of proteins, from averagely 12.9 (control breads) to 29.6% (fortified breads) (17.7–24.7 g/100 g of dry matter respectively), and an improvement in amino acidic profile, with a better balancing of essential amino acids (lysine and sulfur amino acid contents), without affecting protein integrity. Regarding *in vitro* gastro-intestinal digestibility, sample C (19% chickpea proteins) showed the best results, having a comparable protein digestibility to its control bread—48.8 ± 1.1% versus 51.7 ± 2.3%, respectively.

**Conclusion:**

The results showed how the fortification with chickpea and/or pea protein concentrate improved the nutritional profile of bread. These prototypes seem to be a valid strategy to also increase the introduction of high biological value proteins. Furthermore, the not-expected lower digestibility suggested the possible presence of residual anti-nutritional factors in the protein concentrates interfering with protein digestibility. Therefore, it seems of fundamental importance to further investigate these aspects.

## 1 Introduction

The growing concern of consumers for topics such as sustainability and health led food companies to search for new protein sources that can partially or totally substitute animal ones. The biological value of proteins is an important parameter for the classification of protein sources and is evaluated based on the composition of essential amino acids in relation to human needs and the ability of the protein to be digested, absorbed, and retained by the body (1). On one hand, animal proteins ensure a high biological value due to the high presence of essential amino acids and good digestibility, and on the other hand, intensive farming practices bring different problems that are no longer sustainable. This impact on the environment forces to switch toward increasingly plant-based diets (2, 3).

In this scenario, legumes represent a good protein source. Legumes, also called pulses, are characterized by more than 750 *genera* and 16,000–19,000 *species* (4). They are well known for their good nutritional values, showing a good protein content and amino acidic profile (4). In particular, legume content in protein varies from species to species (i.e., peas, beans, chickpeas, lupin, and lentils), ranging from 17 to 46% (5). Their consumption, particularly estimated to a pulses median daily consumption of 52.1 g in the population under investigation, is associated with health benefits such as the increase in satiety, decrease of the post-prandial glycemic index and level of cholesterol, and prevention and/or control of diet-related chronic diseases (i.e., type II diabetes, cardiovascular diseases, and cancer) (6).

Legumes are second only to cereals as a crop worldwide, with peas, chickpeas, lentils, and beans as the most consumed (7). Furthermore, they bring the environmental advantage of having a low impact in terms of CO_2_ emissions and water consumption (4, 8). However, not all the legumes produced worldwide are used for food. Indeed, a portion that ranges from 5 up to 25% of the legumes produced is destined as food waste (9). In particular, from 0.13 million tons of chickpeas and 0.93 million tons of peas harvested per year, averagely 13,000 and 116,000 tons per year are, respectively, wasted even if still usable (10). Usually, these products are wasted for being non-compliant. For this reason, they can be still considered a good source of nutrients such as proteins but also fibers, lipids, and micronutrients (9).

Nevertheless, plant-based proteins are associated with a low biological value and a lower accessibility of proteins (1, 11, 12). There is, however, the possibility of combining different plant-protein sources in order to rebalance the amino acid profile, especially essential amino acids. Indeed, fortified foods play a key role. A fortified food is defined as a food that is added with macronutrients and/or micronutrients through specific technological strategies to enrich its nutritional profile (13). It is a viable technology to reduce malnutrition, both in the case the access to food is limited and also if existing food supplies fail to provide adequate levels of some nutrients in the diet, without drastically changing the usual diet (14).

Bread is a widespread food worldwide, with different formulations depending on the area. It is in itself a food with high energy content and, in particular, carbohydrates, so it lends itself well to fortification with protein-based products (4). The addition of legume flours to bread formulations showed an increased nutritional value of the fortified products (9).

An important nutritional limitation of bread is lysine deficiency (which is an essential amino acid), and the addition of legumes (rich in lysine) to the product could be a valid alternative, more eco-sustainable than animal proteins, to balance the amino acid profile of bread (15, 16). Indeed, the possibility of combining cereal-based with legume-based proteins can have a positive impact on the consumption of high biological value vegetable proteins in the diet (4). Pulses are, indeed, rich in aspartic acid, glutamic acid, leucine, and, especially, lysine but are generally lacking in sulfur amino acids and tryptophan (17). In particular, chickpea are rich in glutamic acid but also in aspartic acid and arginine while are lacking in sulfur amino acids (17). Peas, on the other hand, have a higher variability depending on the ratio between albumins and globulins (influenced by the variety). Generally varieties that are more abundant in albumins are characterized by a higher presence of lysine, sulfur amino acids, threonine, and tryptophan. On the contrary, varieties with greater amounts of globulins are richer in arginine, isoleucine, leucine, and phenylalanine but lack more in sulfur amino acids (17).

Despite what was said above, the high nutritional potential of legumes is often limited by the presence of anti-nutritional factors. This can interfere with the bioaccessibility and digestibility of nutrients, in particular proteins (18, 19). Anti-nutritional factors can be distinguished depending on whether they are protein- or non-protein-based anti-nutritional factors. In particular, non-protein anti-nutritional factors, such as tannins, raffinose, and saponins, can affect the bioaccessibility of some compounds (i.e., iron) or cause damage to the intestinal walls (20, 21), while protein-based anti-nutritional factors, such as lectins, trypsin, chymotrypsin, and amylase inhibitors, can interfere with the action of digestive enzymes, thus leading to reduced digestibility of nutrients (22). Therefore, this study aims to investigate the possible use of protein concentrates, extracted from food wastes (pea and chickpea), in the fortification of different formulations of bread. The authors proceeded with the formulation of various products which were then tested in order to evaluate whether actual use was a valid option to allow the improvement of the nutritional profile of these foods and evaluate the effect of fortification on the protein digestibility of such products.

## 2 Materials and methods

The prototypes of fortified breads analyzed for this study were developed within the European project PROLIFIC (BBI-HORIZON 2020) entitled “*Integrated cascades of processes for the extraction and valorization of proteins and bioactive molecules from legumes, fungi, and coffee agro-industrial side streams*.” This project was carried out with the aim of obtaining protein fractions from industrial residues that can be used in various fields, including the food sector, such as fortifying agents in different food systems.

### 2.1 Raw materials

Chickpeas (*Cicer arietinum L.,* Pascià variety) and peas (*Pisum sativum,* green pea) non-compliant residues were provided by Conserves France (Saint Sylvestre sur Lot, France), and they were sampled both at their French and/or Italian production plants. In particular, chickpeas were harvested in 2018 in Italy and were discarded at selection, after harvesting in the field, due to shape/color defects, while peas were harvested in 2018 in Italy and France and were discarded after blanching process from different companies due to shape/color defects. Production of protein concentrates (60% of protein content) from legumes non-compliant residues was performed by Direct Aqueous Extraction (DAE), as described by Prandi et al. (9), using food wastes as raw materials. In particular, the DAE method was based on the protocol developed by the “*Stazione Sperimentale per l’Industria delle Conserve Alimentari*” (SSICA) (9). In brief, the extraction was carried out using phosphate buffer (0.05 M Na_3_PO_4_ and 0.1 M NaCl) at pH 7.2 and added in 1:2 ratio to the matrix. The extraction was carried out under continuous stirring at room temperature for 3 h. The protein fraction solubilized was then recovered using a decanter. Finally, the proteins were precipitated by acidification at their isoelectric point (pH 4.5) by the addition of 0.1 N HCl and separated from the supernatant by a centrifugation step. The pellets obtained were then freeze-dried in order to obtain the protein concentrate. In the evaluation of the process, the integrity of the proteins, evaluated as DH% and D%, was highly regarded. The protein extracts thus obtained were intended for use as ingredients for new formulations. It was therefore considered important to evaluate the nutritional profile of the final products.

### 2.2 Definition of bread formulations

The samples were produced by the Stolzenberger Bäckerei (Germany) using pea and chickpea-based protein concentrates as fortifying agent. For the purposes of the study, five different formulations of fortified breads, with the respective control breads, were developed and studied. The samples, as shown in Table 1, were formulated adding 19% pea protein isolate (*N* = 2), 19% chickpea protein isolate (*N* = 2), and a 16% mixture of pea and chickpea (in 50:50 ratio) protein isolate (*N* = 1) to different recipes of breads.

**Table 1:** Definition of control and fortified bread formulations.

Control bread			Fortified bread		
Name	Code	List of ingredients	Name	Code	List of ingredients
Spelt bread with walnuts	C A	150 g sourdough from spelt wholemeal flour, 175 g spelt wholemeal flour, 100 g oat flakes, 25 g chia seeds, 175 mL warm water, 10 g salt, 7.5 mL oil, 2.25 g yeast, 15 g walnuts	Spelt bread with **pea**-protein and walnuts (pea concentrate added: 19%)	A	150 g sourdough from spelt wholemeal flour, 175 g spelt wholemeal flour, 150 g pea protein, 100 g oat flakes, 25 g chia seeds, 175 mL warm water, 10 g salt, 7.5 mL oil, 2.25 g yeast, 15 g walnuts
Spelt bread with sunflower seeds	C B	150 g sourdough from spelt wholemeal flour, 175 g spelt wholemeal flour, 100 g oat flakes, 25 g chia seeds, 175 mL warm water, 10 g salt, 7.5 mL oil, 2.25 g yeast, 15 g sunflower	Spelt bread with **pea**-protein and sunflower seeds inside (pea concentrate added: 19%)	B	150 g sourdough from spelt wholemeal flour, 175 g spelt wholemeal flour, 150 g pea protein, 100 g oat flakes, 25 g chia seeds, 175 mL warm water, 10 g salt, 7.5 mL oil, 2.25 g yeast, 15 g sunflower
Multi bread without yeast with rye flakes, oat flakes, sunflower, pumpkin, linseed, honey, water	C C	150 g sourdough, 175 g spelt wholemeal flour, 100 g oat flakes and rye flakes, 25 g chia seeds, 175 mL warm water, 10 g salt, 7.5 mL oil, 3.75 g sunflower seed, 3.75 g pumpkin seed, 3.75 g linseed, 3.75 mL honey water	Multi bread with **chickpea**-protein without yeast with rye flakes, oat flakes, sunflower seed, pumpkin seed, linseed, honey water (chickpea concentrate added: 19%)	C	150 g sourdough, 175 g spelt wholemeal flour, 150 g chickpea protein, 100 g oat flakes and rye flakes, 25 g chia seeds, 175 mL warm water, 10 g salt, 7.5 mL oil, 3.75 g sunflower seed, 3.75 g pumpkin seed, 3.75 g linseed, 3.75 mL honey water
Three-grain bread with rye, wheat, oat	C D	150 g sourdough from spelt wholemeal flour, 175 g spelt wholemeal flour, 100 g oat flakes, 25 g chia seeds, 175 mL warm water, 10 g salt, 7.5 mL oil, 2.25 g yeast	Three-grain bread (grain: rye, wheat, oat) with **chickpea**-protein (chickpea concentrate added: 19%)	D	150 g sourdough from spelt wholemeal flour, 175 g spelt wholemeal flour, 150 g chickpea protein, 100 g oat flakes, 25 g chia seeds, 175 mL warm water, 10 g salt, 7.5 g oil, 2.25 g yeast
Spelt-barley-bread with pumpkin and sesame seeds	C E	125 g sourdough, 150 g spelt wholemeal flour, 125 mL water, 7.5 g linseeds, 7.5 g sunflower seed, 7 g salt, 6.75 mL oil, 2 eggs, 1.85 g yeast	Spelt-barley-bread with **pea**- and **chickpea**- protein with pumpkin and sesame seeds (pea and chickpea concentrate added: 16% in 50:50 ratio)	E	125 g sourdough, 150 g spelt wholemeal flour, 50 g chickpea protein, 50 g pea protein, 125 mL water, 7.5 g linseeds, 7.5 g sunflower seed, 7 g salt, 6.75 mL oil, 2 eggs, 1.85 g yeast

The yeast used was a fresh yeast type “Fala Univrsalhefe” purchased from LESAFFRE Deutschland GmbH (Kehl, KEL, Germany).

### 2.3 Recipe development and baking process

The main objective was to generate artisan-baked goods, having the main product claim “high-protein content.” Therefore, the amount of plant-based proteins added to the samples was chosen to be 19% for chickpea protein and pea protein and 16% for the mixture of them to obtain final products with the 20% of the energy value provided by protein, following current EU legislation (Council Regulation (EC) 1924/2006) (23). The prototypes have been generated after pre-prototype optimizations previously tested. The aim was to optimize the processing conditions by following sourdough technology steps and applying slow baking principle, whereas the intention was not to alter the techno-functional properties of protein and other ingredients used for fortification of the baked goods. For the sourdough production, the “BÖCKER Reinzucht-Sauerteig” (with main lactic acid bacteria contained being *L. sanfranciscensis*) starter culture (Ernst Böcker GmbH Co. & KG, Minden, MI, Germany) was used. Specifically, 1 kg of starter culture has been mixed with 10 kg of the respective flour, and 10 L of water has been added. The “BÖCKER Reinzucht-Sauerteig” starter culture has been used fresh, and the temperature of the sourdough has been set to 28°C, which has been kept for a fermentation time of 15 h. For which concern the fermentation conditions, at the beginning of the sourdough fermentation, a temperature of 28°C has been set and has been kept until the sourdough fermentation was finished. Another aim was to reduce the gluten in the products, which became possible by increasing the level of protein ingredients in the products, using also as bulk base minor cereals and seed ingredients.

Standard baking equipment and machinery has been deployed, including: kneaders—a Diosna SP 160 spiral kneader (Dierks, Osnabrück, OS, Germany) for the sourdough and a Pietroberto Fast 80 spiral kneader (Pietroberto SRL - Piovene Rocchette, VI, Italy) for product kneading—, baking forms, dough rest in proofing chambers (Bongard, Holtzheim, Bas-Rhin, France), and finally slow-baking in MIWE—Roll In baking ovens (Miwe GmbH, Arnstein, MSP, Germany). However, production conditions have been adjusted in order to be able to profit from the advantages of sourdoughs, as shown in Supplementary Figure S1, such as sourdoughs, pre-doughs, and sourdough-starters.

Several recipes have been subjected to baking optimization trials. Following technology transfer procedures, the best performing ones have been selected, improved, and validated. Final recipes are shown in Table 1. All the breads were made of spelt wholemeal flour and sourdough and added with cereals, seeds, and nuts. The only sample having egg as ingredient was sample E.

After recipe formulation, the bulk ingredients (wholemeal flour, water) have been mixed with the food-grade additives (e.g., yeast, salt, oil, eggs, PROLIFIC protein extracts, and different seeds). The technological procedures consisted of kneading, dough rest, followed by processing into bread forms and rest in the fermentation chamber at high chamber humidity. Doughs have also rested overnight using the slow baking method (reduced yeast or only made with sourdough) and baked the next day. The baking process was non-linear, meaning first phase with higher temperatures, with automatic temperature drop to lower ones. During the baking process, steam has also been added automatically.

Since water absorption and baking quality vary even within the sourdough conditions, thus an adjustment of dough yield has been performed in order to obtain balanced crumb/crust ratios (Supplementary Figure S2).

In general, modified standard baking trials were carried out as closely as possible to the baking test developed by the Max Rubner Institute (MRI) for wholemeal flours. The baking trials were performed in loaf pans with a significantly greater amount of dough using conventional technology (laboratory kneader and manual preparation). All the samples underwent the same baking conditions at 230°C for 30 min. The advantage of this baking is that it incorporates a greater quantity of water (flour–water ratio) into the recipe.

### 2.4 Proximate analysis

#### 2.4.1 Dry matter

The dry matter was estimated following a standard procedure (24), placing 1 g of each bread at 104°C in a 1,060 ventilated oven (Memmert GmbH, Schwabach, SC, Germany) until weight stability (approximately 23 h). The analysis was conducted in duplicate.

#### 2.4.2 Ashes

Ashes were determined following a standard procedure (25), placing 1 g of each bread in proper crucibles at 550°C in a muffle-type furnace until completely incinerated (approximately 5 h). The analysis was conducted in duplicate.

#### 2.4.3 Protein

Total nitrogen content was determined performing the Kjeldahl method according to the European Regulation (UE) n. 152/2009 (26) using a DK 8 digestion unit and a UDK semi-automatic distillation unit (VELP Scientifica S.r.l., Usmate Velate, MB, Italy). In brief, 1 g of sample was digested with 17 mL of sulfuric acid 96%, copper(II)oxide, 1 tablet of catalyst, and 1 tablet of defoamer (VWR International S.r.l., Radnor, PA, United States) for 1 h at 420°C. Samples were then distilled with 32% NaOH (Sigma–Aldrich Co., St. Louis, MO, United States), and ammonia was collected in the toning solution—4% borate acid, methyl red, and bromocresol green 1 (mg/ml in methanol) (Sigma–Aldrich Co., St. Louis, MO, United States). The titration was performed using 0.1 N HCl (PanReac AppliChem, Darmstadt, DA, Germany). Being the samples made of a mixture of different protein containing ingredients, the standard conversion factor of 6.25 was used for the calculation of the total protein content (27). The analysis was conducted in duplicate.

#### 2.4.4 Total fat

Total fat content was estimated with the Soxhlet method following the AOAC standard procedure (28) using a SER 148/3 semi-automatic Soxhlet extractor (VELP Scientifica S.r.l., Usmate Velate, MB, Italy) and diethyl ether (Millipore Corporation, Burlington, MA, United States) as extraction solvent. The analysis was conducted in duplicate.

#### 2.4.5 Fibers

Total dietary fibers (soluble and insoluble) were determined following the AOAC standard procedure (29). The analysis was conducted in duplicate.

#### 2.4.6 Salt

The salt content was estimated from the recipe.

#### 2.4.7 Total carbohydrates

Total carbohydrates were calculated as a difference by the rest of the nutrients following the equation below:


$$TC=100-\left(W-A-TF-F-P\right)$$

where *TC* stands for the total carbohydrates, *W* for water, *A* for ashes, *TF* for total fat, *F* for fiber, and *P* for protein.

### 2.5 Protein identification

#### 2.5.1 Extraction of proteins and quantification

The protein fraction of samples was extracted adding 1 mL of extraction buffer (4 M urea, 100 mM NH_4_HCO_3_, and 5 mM DTT) to 100 mg of sample. Samples were extracted for 90 min at room temperature under agitation (30). After extraction, samples were centrifuged (20,817 *g*, 4°C, 15 min), and the supernatant was filtered with 45 μm nylon filters.

The protein content of the clear supernatants was estimated using a Qubit Fluorometer^™^ with the Quant-iT Protein Assay Kit (Invitrogen, Carlsbad, CA, United States), following the guidance material.

#### 2.5.2 SDS-PAGE analysis

The electrophoretic separation on polyacrylamide gel was performed using a Criterion XT Bis-Tris Gel at 10% (BIO-RAD, Hercules, CA, United States). The procedure was carried out as described by Prandi et al. (9). In brief, the volume corresponding to 40 μg of protein, estimated with Qubit Fluorometer^™^, for each sample was transferred to suitable Eppendorf tubes. Samples were dried under nitrogen, reconstituted with 25 μL of sample buffer—17.5 μL water, 6.25 μL buffer 6x, and 1.25 μL reducing agent 2x (BIO-RAD, Hercules, CA, United States)—and thermocycled for 5 min at 95°C. After cooling down to room temperature, samples and protein marker (Standard Precision Plus ProteinTM - BIO-RAD, Hercules, CA, United States) were loaded onto the gel. The run was set at 150 V for 45 min using a PowerPac^™^ universal power supply (BIO-RAD, Hercules, CA, United States). Finally, gel was colored with a staining solution—50% water, 40% methanol, 10% glacial acetic acid, and 1 g/L Coomassie Brilliant Blue—and destained with consequential washing with destaining solution—50% water, 40% methanol, and 10% glacial acetic acid. The gel was scanned using a GS-800 calibrated imaging densitometer (BIO-RAD, Hercules, CA, United States).

The identification of proteins was then performed for comparison with the literature (9).

### 2.6 Amino acidic profile

#### 2.6.1 Total amino acid determination

The determination of total amino acids was performed, as described by Prandi et al. (9), with an acid hydrolysis. In brief, 500 mg of sample was weighted in Pyrex glass tubes with teflon-lined screw caps, added with 6 N HCl, and hydrolyzed for 23 h at 110°C. After the hydrolysis, samples were cooled at room temperature, added with 0.75 mL of internal standard (nor-leucine 50 mM in 0.1 N HCl), and filtered. The clear solutions were brought to 250 mL with MilliQ^®^ water (Millipore Corporation - Burlington, MA, United States). For the determination of cysteine and methionine, a pre-oxidation is needed. For this reason, prior to the acid hydrolysis, samples for the determination of these two amino acids were added with 2 mL of performic acid freshly prepared (performic acid 95% and H_2_O_2_ in ratio 90:10). After 16 h at 0°C, the reaction was stopped with the addition of 0.3 mL of bromidic acid, and samples were flushed with nitrogen. When dried, samples underwent the acid hydrolysis. The analysis was conducted in duplicate.

For the determination of a calibration, standard solution was made mixing a 2.5 mM standard mixture of amino acids (Thermo Scientific, Waltham, MA, United States) with a mixture of amino acids, 2.5 mM each (nor-leucine, cysteic acid, and methionine sulfone), in a ratio of 1:1. By sequential dilution, the different concentration points were obtained: 1.25 mM, 0.625 mM, 0.3125 mM, 0.156 mM, and 0.078 mM.

Hydrolyzed samples and standards were derivatised using the AccQ-Fluor reagent kit (Waters, Milford, MA, United States) and kept for the further analysis.

#### 2.6.2 Tryptophan determination

The tryptophan determination was performed with an alkaline hydrolysis as described by Cutroneo et al. (30). In brief, 0.15 g of sample was weighted in Pyrex glass tubes with Teflon-lined screw caps and added with 4 N NaOH and 0.15 mL of internal standard (α-methyl-tryptophan 50 mg/100 mL in deionized water). The hydrolysis was performed at 100°C for 6 h, and samples were then cooled to room temperature. Finally, samples were centrifuged (3,220 *g*, 4°C, 45 min), filtered with 0.45 μm nylon filters, and brought up to 10 mL in volumetric flask with deionized water. The analysis was conducted in duplicate.

#### 2.6.3 UPLC-ESI-MS analysis

Both acid and alkaline-hydrolyzed samples were analyzed using a UPLC ACQUITY system coupled with an ACQUITY SQ ESI-MS system (Waters, Milford, MA, United States). The analysis was performed with an ACQUITY UPLC Peptide BEH C18 (300 Å, 1.7 μm, 2.1 mm 170 × 150 mm) column (Waters, Milford, MA, United States) and an ACQUITY UPLC Peptide BEH C18 VanGuard^™^ (300 Å, 1.7 μm, 2.1 mm × 5 mm) pre-column (Waters, Milford, MA, United States).

The analysis was performed as reported in the literature by Buhler et al. (31). The acquisition was performed in SIR mode.

Data acquisition and processing were performed with MassLynx^™^ V4.0 (Waters, Milford, MA, United States).

#### 2.6.4 Amino acidic score estimation

The amino acidic score of each sample was estimated for each essential amino acid as the ratio between the mg/g of protein and the mg/g of protein required for the diet. The reference values were collected from the requirements set by FAO for children (3–18 years old) and adults (32).

### 2.7 Protein integrity

#### 2.7.1 Degree of hydrolysis

The degree of hydrolysis (DH%) of samples was estimated extracting the protein fraction and subsequentially performing the OPA method. The analysis was conducted in duplicate.

The extraction of the protein fraction was performed as previously described in Section 2.5.1. The extracted samples were then filtered and properly diluted. The DH% was estimated performing the OPA method in accordance with the procedure reported by Spellman et al. (33). The absorbance was measured at 340 nm with a JASCO B-530 UV–VIS spectrophotometer (JASCO, Oklahoma City, OK, United States). A calibration curve, using isoleucine as standard, was also performed: 2 mg/mL, 1 mg/mL, 0.5 mg/mL, 0.25 mg/mL, and 0.125 mg/mL. The DH% was calculated as the ratio between the free amino groups determined with the calibration curve and the total amino groups of the samples.

#### 2.7.2 Enantiomeric purity

The enantiomeric purity (D-enantiomers %) was determined of samples following a standard procedure (30). In brief, 0.5 g of each sample was added with 5 mL of 6 N HCl and hydrolyzed for 6 h at 110°C. Then, samples were cooled to room temperature, filtered, and derivatised first in HCl 2 N in 2-propanol (90°C for 1 h) and then with dichloromethane and trifluoroacetic anhydride (50°C for 30 min). The analysis was conducted in duplicate.

After drying under nitrogen flow, samples were resuspended in dichloromethane and analyzed with an Agilent Technologies 7820A gas chromatograph (Agilent Technologies, Palo Alto, CA, United States) and coupled to an Agilent Technologies 5977B mass spectrometer (Agilent Technologies, Palo Alto, CA, United States). The acquisition was performed in SIR mode.

### 2.8 Protein digestibility

#### 2.8.1 *In vitro* gastro-intestinal digestion protocol

All bread samples were subjected to simulated *in vitro* gastro-intestinal digestion procedure following the static harmonized INFOGEST procedure reported by Brodkorb et al. (34). The analysis was performed using an ES-20 orbital shaker-incubator (SIA BioSan, Riga, LV, Latvia). In brief, 1 g of grinded sample was added with pH 6 simulated saliva (containing porcine α-amylase, 75 U/mL) in a ratio of 1:1 w/v to form the bolus. After 2 min at 37°C and 180 rpm, the bolus was added with the pH 3 simulated gastric fluid (containing porcine pepsin, 2000 U/mL) in a ratio of 1:1 v/v to form the chyme. After 2 h at 37°C and 180 rpm, the chyme was added with the pH 8 simulated intestinal fluid (containing pancreatin, 100 U/mL of trypsin, and bile salts, 10 mM) in a ratio of 1:1 v/v to form the chyle. After 2 h at 37°C and 180 rpm, the enzymes were inactivated thermically boiling samples at 90°C for 15 min. The pH was checked and adjusted during all the phases. Samples were centrifuged at 4°C and 3,220 g for 45 min, and the supernatant was saved for the further analysis. The analysis was conducted in duplicate.

Two different blanks were also determined, one using simulated fluids with no enzyme and one using deionized water instead of the sample.

#### 2.8.2 Degree of hydrolysis of digested samples

The DH% of the digested samples was estimated using the OPA method, as previously described in Section 2.7.1, by properly diluting the digestates. The analysis was conducted in duplicate.

### 2.9 Statistical analysis

**Figure 1:**
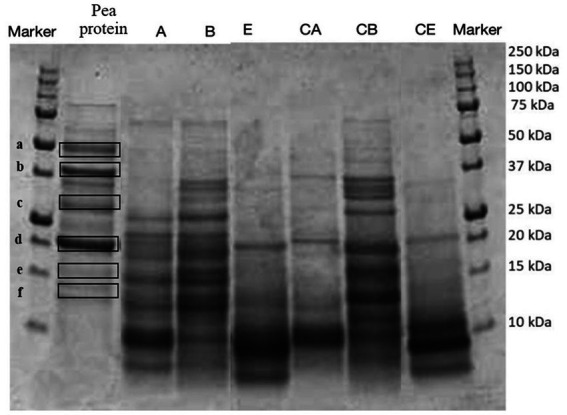
SDS-PAGE analysis of the protein profile of fortified and control breads compared with the extracted pea protein concentrate. Samples coding: (A) Spelt bread with pea protein and walnuts (19% pea concentrate) and its control C A; (B) Spelt bread with pea protein and sunflower seeds inside (19% pea concentrate) and its control C B; (E) Spelt-barley-bread with pea and chickpea protein with pumpkin and sesame seeds (16% pea and chickpea concentrate in 50:50 ratio) and its control C E.

**Figure 2:**
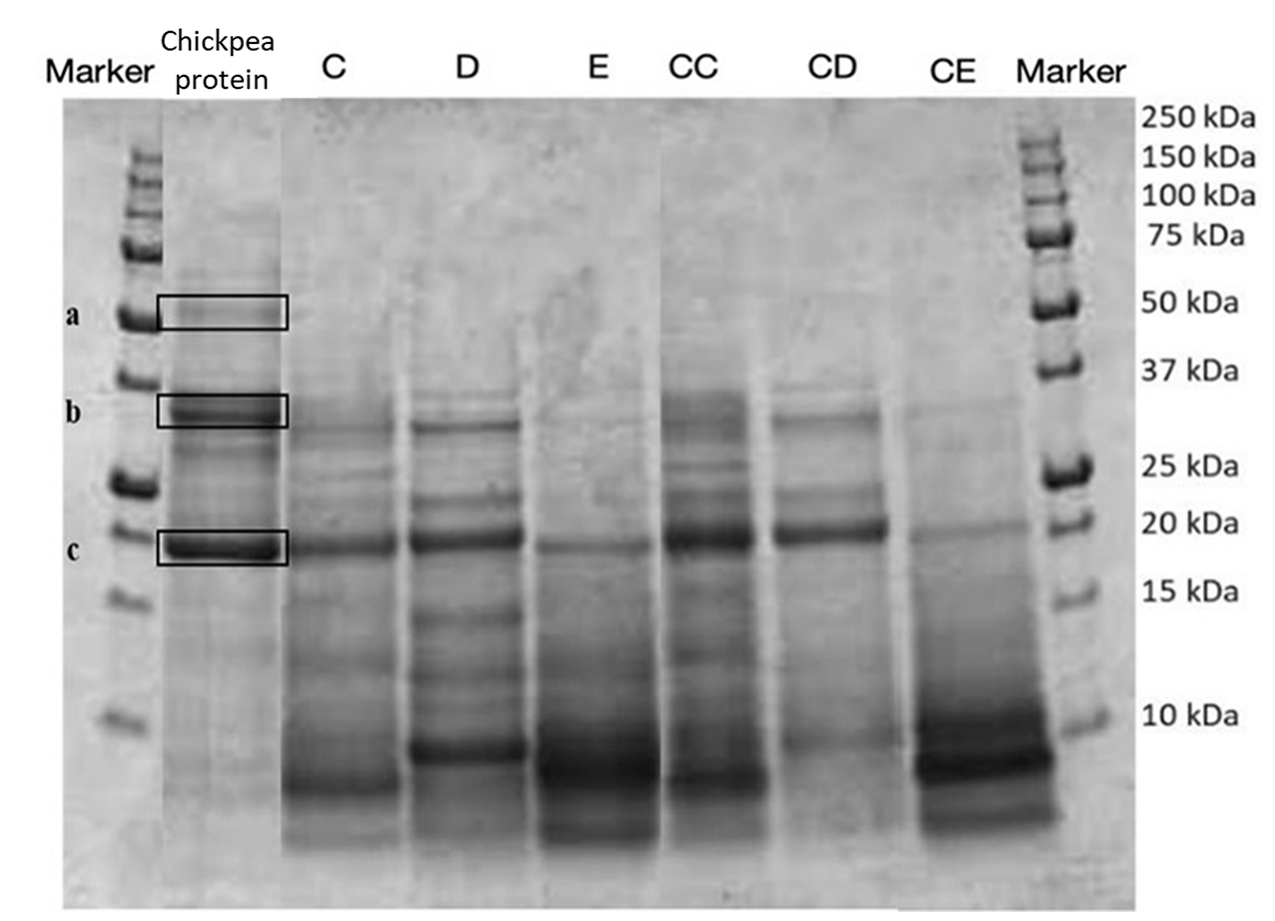
SDS-PAGE analysis of the protein profile of fortified and control breads compared with the extracted chickpea protein concentrate. Sample coding: (C) Multi bread with chickpea protein without yeast with cereal flakes and seeds (19% chickpea concentrate) and its control C C; (D) Three-grain bread (rye, wheat, and oat) with chickpea protein (19% chickpea concentrate) and its control C D; (E) Spelt-barley-bread with pea and chickpea protein with pumpkin and sesame seeds (16% pea and chickpea concentrate in 50:50 ratio) and its control C E.

**Figure 3:**
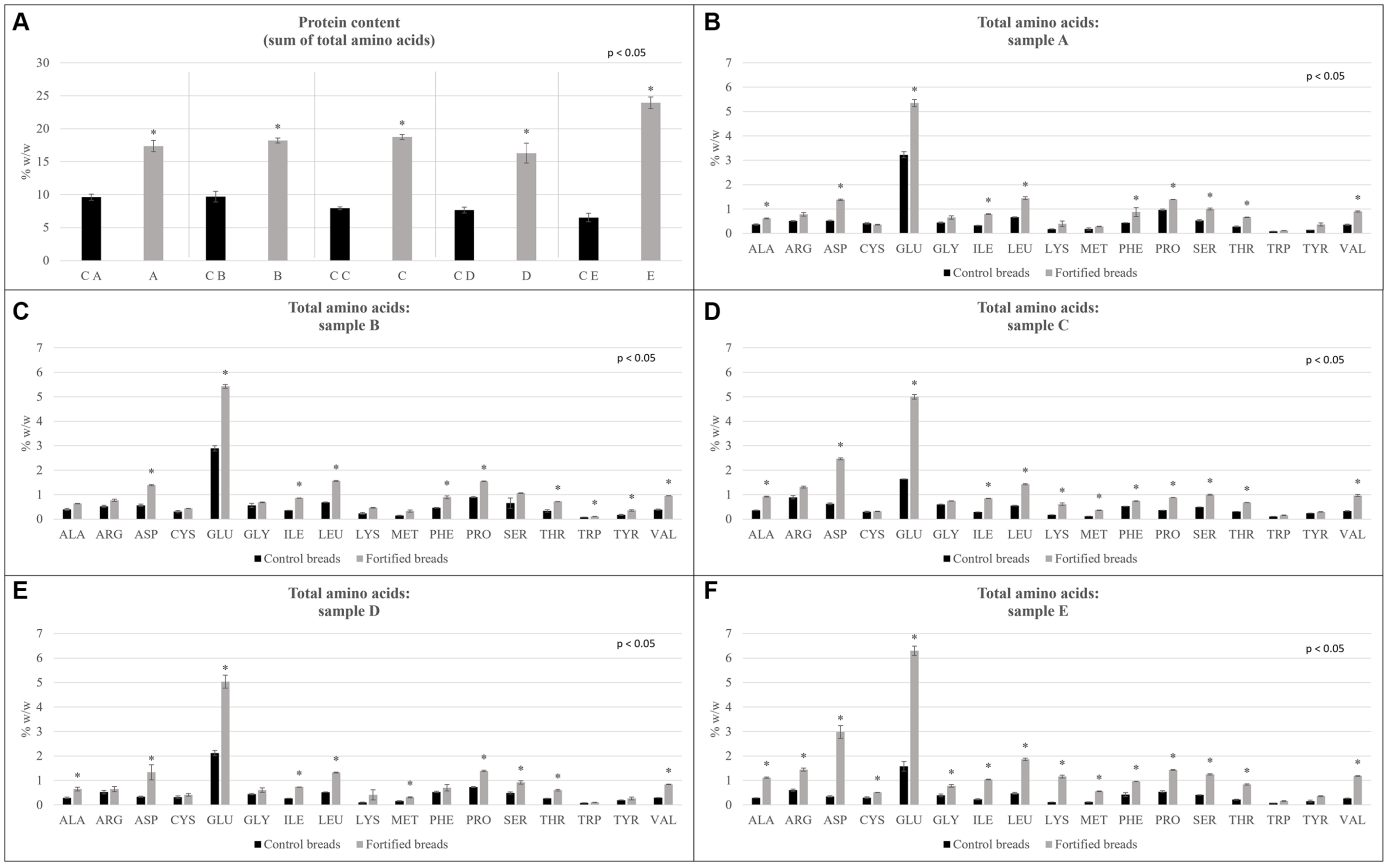
Total amino acids determined for samples showed for control breads (in black) and fortified breads (in gray). **(A)** Protein content reported as the sum of total amino acids. **(B–F)** Amino acidic profile of samples. Sample coding: (A) Spelt bread with pea protein and walnuts (19% pea concentrate) and its control C A; (B) Spelt bread with pea protein and sunflower seeds inside (19% pea concentrate) and its control C B; (C) Multi bread with chickpea protein without yeast with cereal flakes and seeds (19% chickpea concentrate) and its control C C; (D) Three-grain bread (rye, wheat, and oat) with chickpea protein (19% chickpea concentrate) and its control C D; (E) Spelt-barley-bread with pea and chickpea protein with pumpkin and sesame seeds (16% pea and chickpea concentrate in 50:50 ratio) and its control C E. The asterisk in figures reports the significant differences (ρ < 0.05) between control and fortified bread determined with the paired *T*-test.

**Figure 4:**
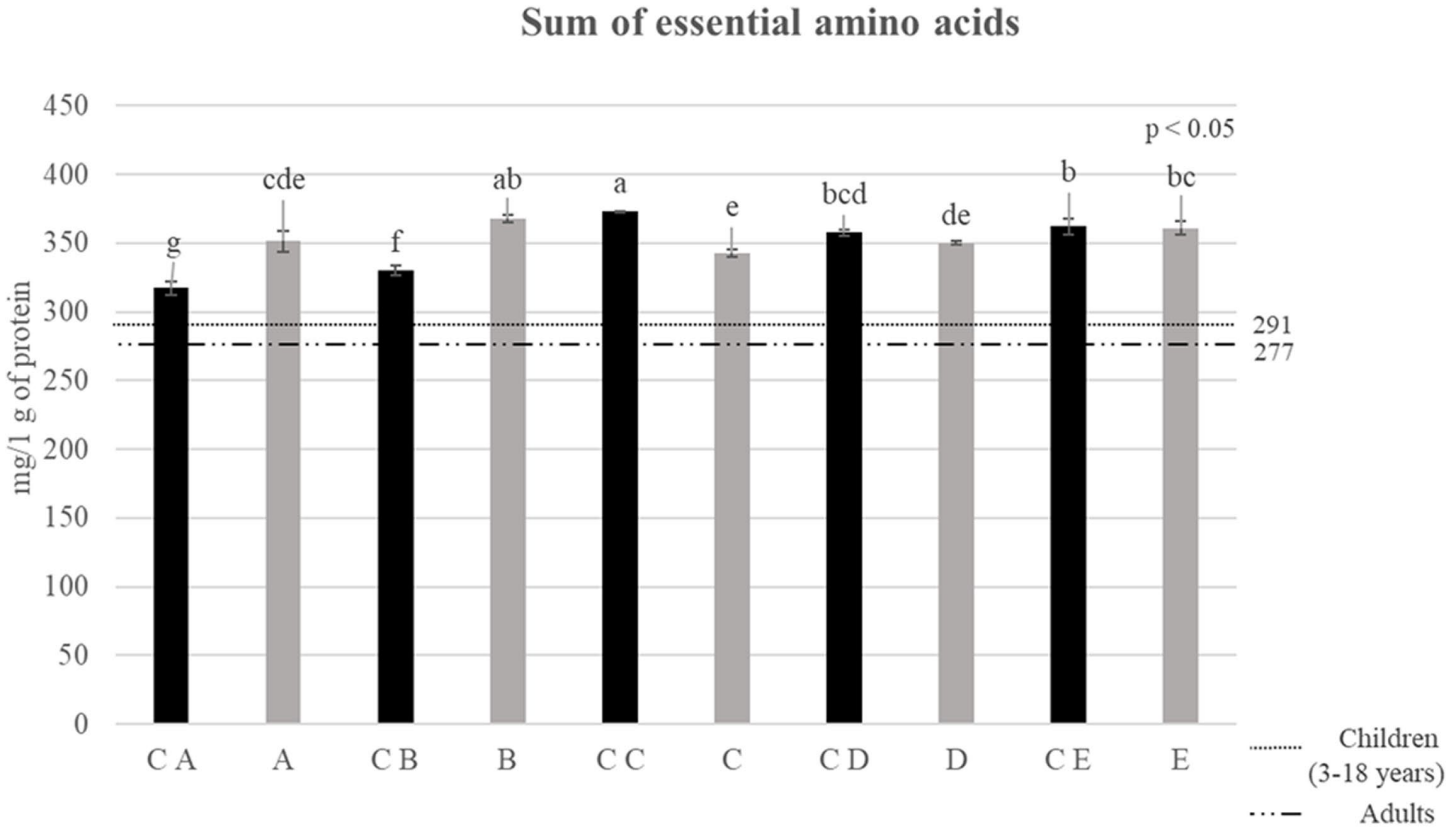
Sum of essential amino acids reported for control breads (in black) and fortified breads (in gray) expressed as mg/1 g of protein. Samples coding: (A) Spelt bread with pea protein and walnuts (19% pea concentrate) and its control C A; (B) Spelt bread with pea protein and sunflower seeds inside (19% pea concentrate) and its control C B; (C) Multi bread with chickpea protein without yeast with cereal flakes and seeds (19% chickpea concentrate) and its control C C; (D) Three-grain bread (rye, wheat, and oat) with chickpea protein (19% chickpea concentrate) and its control C D; (E) Spelt-barley-bread with pea- and chickpea-protein with pumpkin and sesame seeds (16% pea and chickpea concentrate in 50:50 ratio) and its control C E. The reference lines in the graph refer to the requirements set by FAO for children (3–18 years) and adults (32). Letters refers to significant differences between samples (ρ < 0.05) determined with one-way ANOVA.

Statistical analysis was performed using SPSS Statistic version 26.0 (Statistical Package for Social Science, Chicago, IL, United States). To investigate the differences in the sum and profile of total amino acids (Figure 1), the DH% (Figure 2), the protein content based on the determination method (Table 2), and the D% (Table 3) between samples and its relative control, paired *T*-test was performed (ρ < 0.05), while, to investigate differences in total fat and protein content (Table 4), the sum of EAA (Figure 3), and the DH% of digestate (Figure 4) among all samples, one-way ANOVA was performed (ρ < 0.05).

**Table 2:** Proximate composition of fortified and control breads expressed on the dry matter.

			Composition (g/100 g of dry matter)					
	Codes	Dry matter	Ash	Total fat	Total carbohydrates	Fiber	Protein	Salt
Control breads	C A	82.4	3.3	17.7	51.3	7.8	18.4	1.6
	C B	76.5	3.5	20.5	50.9	4.8	18.6	1.7
	C C	69.4	4.1	32.7	37.5	6.9	17.0	1.9
	C D	71.6	3.8	7.0	61.7	8.8	16.9	1.8
	C E	64.7	4.4	12.4	57.8	5.6	17.8	2.0
Fortified breads	A	96.4	3.7	18.0	47.5	5.5	23.9	1.3
	B	96.3	4.3	8.0	58.0	4.3	24.2	1.4
	C	93.3	4.0	39.3	24.8	6.2	24.3	1.4
	D	95.6	3.6	8.7	56.6	8.0	21.7	1.4
	E	95.3	4.3	18.5	41.4	4.9	29.6	1.4

Samples coding: (A) Spelt bread with pea-protein and walnuts (19% pea concentrate) and its control C A; (B) Spelt bread with pea-protein and sunflower seeds inside (19% pea concentrate) and its control C B; (C) Multi bread with chickpea-protein without yeast with cereal flakes and seeds (19% chickpea concentrate) and its control C C; (D) Three-grain bread (rye, wheat, oat) with chickpea-protein (19% chickpea concentrate) and its control C D; (E) Spelt-barley-bread with pea- and chickpea- protein with pumpkin and sesame seeds (16% pea and chickpea concentrate in 50:50 ratio) and its control C E.

**Table 3:** Detailed total fat and protein information expressed as g/100 g of dry matter (mean ± st. dev.).

			Composition (g/100 g of dry matter)	
	Codes	Dry matter	Total fat	Protein
Control breads	C A	82.4	17.7 ± 0.64^c^	18.4 ± 0.46^c^
	C B	76.5	20.5 ± 1.24^c^	18.6 ± 0.39^c^
	C C	69.4	32.7 ± 1.32^b^	17.0 ± 0.37^d^
	C D	71.6	6.98 ± 0.59^e^	16.9 ± 0.30^d^
	C E	64.7	12.4 ± 0.55^d^	17.8 ± 0.21^cd^
Fortified breads	A	96.4	18.0 ± 0.06^c^	23.9 ± 0.93^b^
	B	96.3	7.95 ± 0.44^e^	24.2 ± 0.88^b^
	C	93.3	39.3 ± 0.05^a^	24.3 ± 0.81^b^
	D	95.6	8.66 ± 0.14^e^	21.7 ± 0.19^c^
	E	95.3	18.5 ± 0.01^c^	29.6 ± 0.26^a^

Samples coding: (A) Spelt bread with pea-protein and walnuts (19% pea concentrate) and its control C A; (B) Spelt bread with pea-protein and sunflower seeds inside (19% pea concentrate) and its control C B; (C) Multi bread with chickpea-protein without yeast with cereal flakes and seeds (19% chickpea concentrate) and its control C C; (D) Three-grain bread (rye, wheat, oat) with chickpea-protein (19% chickpea concentrate) and its control C D; (E) Spelt-barley-bread with pea- and chickpea- protein with pumpkin and sesame seeds (16% pea and chickpea concentrate in 50:50 ratio) and its control C E. Letters in columns refer to the significant differences (ρ < 0.05) between the nutrient content of all samples performed with one-way ANOVA.

**Table 4:** Protein identification reported for extracted pea protein concentrate performed for comparison with literature (9).

Band code	n° UNIPROT	MW (Da)	Protein	Organism
a	P13918	52,231	Vicilin	*Pisum sativum*
b	Q9T0P5	58,789	LegA class	*Pisum sativum*
c	D3VND9	49,515	Vicilin 47 k	*Pisum sativum*
d	D3VNE2	49,664	Vicilin 47 k	*Pisum sativum*
e	P08688	26,238	Albumine-2	*Pisum sativum*
f	P08688	26,238	Albumine-2	*Pisum sativum*

## 3 Results

### 3.1 Samples description

In this study, different formulations of breads added with extracted proteins from legumes and their respective control breads were analyzed and compared. The main features of the protein concentrates used for the fortification (pea and chickpea) are well elucidated in a previous study by Prandi et al. (9).

The samples, as graphically shown in Supplementary Figure S3 and presented in Table 1 (Section 2.2), are 10, five fortified breads and five control breads. Specifically, samples A and C A are spelt breads with walnuts inside, while in samples B and C B, also spelt bread, we find sunflower seeds inside. Sample C and control C C are particular as they are the only bread without yeast and are made with rye flakes, oat flakes, sunflower and pumpkin seeds, linseed, and honey. Sample D and the related control C D are three-cereal bread with rye, wheat, and barley, and samples E and C E are spelt and barley bread with pumpkin seeds and sesame.

From a compositional point of view, the differences between fortified breads and controls concern the addition of the protein concentrates in the formulation, as stated previously. In fact, in the formulation of breads A and B, we find 19% pea protein concentrates, 19% chickpea protein concentrates in samples C and D, and finally, in sample E, there is a combination of pea protein extracts and chickpeas (16% in total).

### 3.2 Proximate composition

The composition of samples is shown below in Table 5, while more detailed information on total fat and protein content is shown in Table 4.

**Table 5:** Protein identification reported for extracted chickpea protein concentrate performed for comparison with literature (9).

Band code	n° UNIPROT	MW (Da)	Protein	Organism
a	A0A1S2Y087	69,392	Vicilin-like	*Cicer arietinum*
b	A0A1S3E1A0	52,098	Vicilin-like	*Cicer arietinum*
c	A0A1S2XVG1	60,371	Legumin J-like	*Cicer arietinum*

From the data shown in Table 4, it can be observed that there is a higher (ρ < 0.05) protein content in the fortified samples than in the control ones. In particular, the most significant increase in this sense can be found in sample E within which, with the fortification process, it went from 17.8 g of proteins (evaluated on 100 g of dry substance) in the control to 29.6 g/100 g in fortified bread.

The increase in the protein content follows a decrease in the total carbohydrate content (Table 5) that varies from 7.4 (in the case of sample A) to 33.9% (sample C) decrease. The only exception in this sense can be found in sample B, and there was an increase in total carbohydrates from 50.9 in the control to 58.0 g/100 g in the corresponding fortified bread due to a substantial decrease in the lipid fraction.

Regarding the fiber content, there were no particular differences between the controls and the fortified breads, a result in agreement with what was expected. By analyzing the lipid content (Table 4) of the samples, we can state that sample C shows a much higher value of fats than the other products under examination, having 32.7 g/100 g of total lipids within its composition.

### 3.3 Protein profile identification

**Figure 5:**
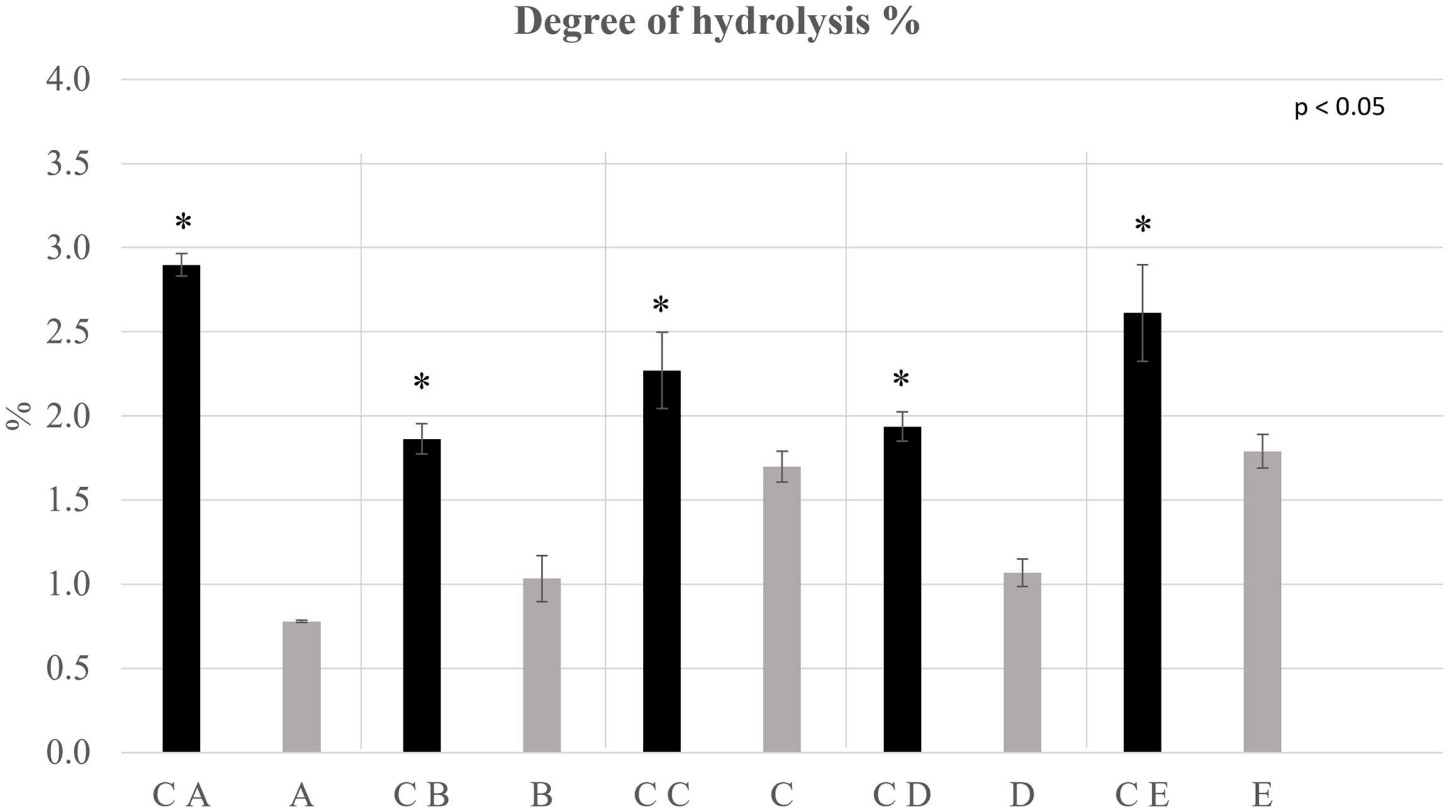
Degree of hydrolysis % determined on the extracted protein fraction of control breads (in black) and fortified breads (in gray). Sample coding: (A) Spelt bread with pea protein and walnuts (19% pea concentrate) and its control C A; (B) Spelt bread with pea protein and sunflower seeds inside (19% pea concentrate) and its control C B; (C) Multi bread with chickpea protein without yeast with cereal flakes and seeds (19% chickpea concentrate) and its control C C; (D) Three-grain bread (rye, wheat, and oat) with chickpea protein (19% chickpea concentrate) and its control C D; (E) Spelt-barley-bread with pea- and chickpea-protein with pumpkin and sesame seeds (16% pea and chickpea concentrate in 50:50 ratio) and its control C E. The asterisks refer to the significant differences (ρ < 0.05) between each sample and its control performed with paired *T*-test.

**Figure 6:**
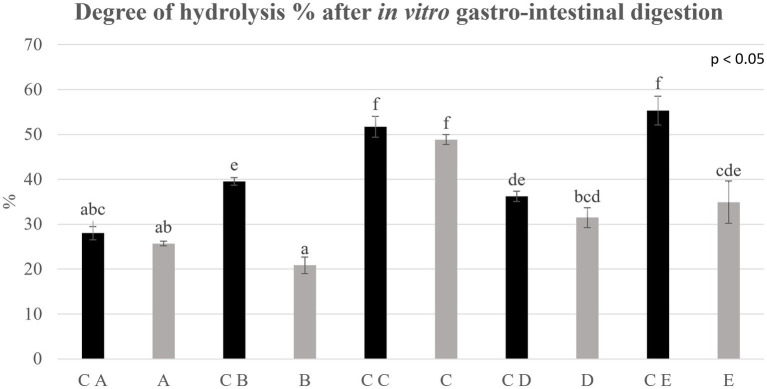
Degree of hydrolysis % determined on control breads (in black) and fortified breads (in gray) digestates. Samples coding: (A) Spelt bread with pea protein and walnuts (19% pea concentrate) and its control C A; (B) Spelt bread with pea protein and sunflower seeds inside (19% pea concentrate) and its control C B; (C) Multi bread with chickpea protein without yeast with cereal flakes and seeds (19% chickpea concentrate) and its control C C; (D) Three-grain bread (rye, wheat, and oat) with chickpea protein (19% chickpea concentrate) and its control C D; (E) Spelt-barley-bread with pea- and chickpea-protein with pumpkin and sesame seeds (16% pea and chickpea concentrate in 50:50 ratio) and its control C E. Letters refer to significant differences (ρ < 0.05) performed with one-way ANOVA.

To identify the proteins present in the protein extracts, an electrophoretic separation was conducted under reducing conditions (SDS-page analysis). As shown in Figures 5, 6, gels obtained from the analysis of fortified breads, control breads, and protein concentrates of peas and chickpeas, respectively, are shown. Since the protein profile shown was very complex, especially given the combination of proteins from cereals and oilseeds and protein concentrates, in both cases (fortification with pea and chickpea protein concentrates), we proceeded to identify the bands for comparison with the literature. From the profile obtained, the bands reported by the protein concentrates used for fortification were identified by comparison with the previous study by Prandi et al. (9).

In Figure 5, the presence of six highlighted bands can be noticed in the pea protein concentrate. The identification of the bands, as presented in Table 6, showed the presence of three principal classes of proteins, namely, vicilins, legumins, and albumins.

**Table 6:** Protein content determined with Kjeldahl method and as the sum of total amino acids expressed as g of protein/100 g if dry matter (mean ± st. dev.).

Protein fraction					
Control breads			Fortified breads		
Code	Kjeldahl method	Total amino acids	Code	Kjeldahl method	Total amino acids
C A	18.3 ± 0.38^*^	11.6 ± 0.45	A	23.9 ± 0.89^*^	18.0 ± 0.86
C B	18.6 ± 0.30^*^	12.6 ± 0.80	B	24.2 ± 0.85^*^	18.9 ± 0.36
C C	17.0 ± 0.26^*^	11.3 ± 0.21	C	21.8 ± 0.76^*^	20.1 ± 0.38
C D	16.9 ± 0.22^*^	10.6 ± 0.45	D	21.8 ± 0.18^*^	17.0 ± 1.50
C E	17.8 ± 0.14^*^	10.0 ± 0.64	E	29.6 ± 0.25^*^	25.1 ± 0.84

Samples coding: (A) Spelt bread with pea-protein and walnuts (19% pea concentrate) and its control C A; (B) Spelt bread with pea-protein and sunflower seeds inside (19% pea concentrate) and its control C B; (C) Multi bread with chickpea-protein without yeast with cereal flakes and seeds (19% chickpea concentrate) and its control C C; (D) Three-grain bread (rye, wheat, oat) with chickpea-protein (19% chickpea concentrate) and its control C D; (E) Spelt-barley-bread with pea- and chickpea- protein with pumpkin and sesame seeds (16% pea and chickpea concentrate in 50:50 ratio) and its control C E. Asterisks in the table refer to the significant differences (ρ < 0.05) between the protein content of each sample determined with the two different methods performed with pair *T*-test.

In Figure 6, the presence of three bands can be noticed for the chickpea protein concentrate. The identification of the bands, as presented in Table 7, showed the presence of three principal classes of proteins, such as vicilin-like and legumin-like proteins.

**Table 7:** Amino acid score reported for lysine (LYS) and sulphur amino acids (SAA, as sum of cysteine and methionine) reported for control and fortified breads compared to the requirements set by FAO for children (3–18 years) and adults (32).

Amino acid score						
	Control breads			Fortified breads		
	Code	LYS	SAA	Code	LYS	SAA
Children (3–18 years)	C A	0.38	2.65	A	0.47	1.60
	C B	0.51	2.04	B	0.53	1.83
	C C	0.46	2.27	C	0.68	1.58
	C D	0.27	2.69	D	0.50	1.94
	C E	0.36	2.77	E	1.01	1.95
Adults	C A	0.41	2.78	A	0.50	1.67
	C B	0.54	2.14	B	0.57	1.91
	C C	0.49	2.38	C	0.73	1.65
	C D	0.29	2.81	D	0.54	2.02
	C E	0.39	2.90	E	1.08	2.04

Samples coding: (A) Spelt bread with pea-protein and walnuts (19% pea concentrate) and its control C A; (B) Spelt bread with pea-protein and sunflower seeds inside (19% pea concentrate) and its control C B; (C) Multi bread with chickpea-protein without yeast with cereal flakes and seeds (19% chickpea concentrate) and its control C C; (D) Three-grain bread (rye, wheat, oat) with chickpea-protein (19% chickpea concentrate) and its control C D; (E) Spelt-barley-bread with pea- and chickpea- protein with pumpkin and sesame seeds (16% pea and chickpea concentrate in 50:50 ratio) and its control C E.

In both cases, by comparison, it is possible to identify the proteins, which are found in the extracted raw materials and the fortified products.

### 3.4 Determination of the protein content

The determination of the protein content in each sample was carried out using the Kjeldahl method, as already described in the methodology section (Section 2.4.3). This type of analysis allows to determine the total nitrogen present in the matrices and, subsequently, allows the calculation of the total protein content using an experimentally determined conversion factor, which is specific to each food. In this specific case, being the samples produced from various protein-containing ingredients, the generic conversion factor of 6.25 was used (27). In fact, the proteins present in the formulations derive from different sources, such as cereals, seeds, and legumes. For this reason, the protein content of the samples was also determined as the sum of the total amino acids, which was quantified as described in the methodology section (Section 2.6). The results of these analyses are therefore presented and compared in Table 2.

From the results reported in the table above, a significant difference can be noticed when comparing the protein content values determined with the Kjeldahl method and the values estimated with the sum of total amino acids. This indicates an overestimation in the protein content of the values determined with the total nitrogen analysis.

### 3.5 Amino acidic profile

The amino acidic profile determined for the samples is shown in Figure 1.

In particular, as shown in Figure 1 panel A, the protein content estimated as the sum of total amino acids, and the comparison between each fortified bread and its control is shown. Analyzing the graph, it can be noticed how, in all cases, there are significant differences between the controls and the related fortified breads (ρ < 0.05), observing indeed an increase in protein content for all fortified samples. In particular, sample E showed triple protein content compared with the control bread. This result confirmed what observed also with the proximate analysis.

Going into more detail, Figure 1 shows, from panel B to panel F, the amino acid profile for each control bread, fortifying bread pair. For all samples, a significant increase (ρ < 0.05) was observed in the content of almost all amino acids, characterizing the protein fraction.

After confirming the general increase in the amino acid content, it is important to evaluate the content of Essential Amino Acids (EAAs) (phenylalanine, isoleucine, histidine, leucine, lysine, methionine, threonine, tryptophan, and valine). The results of this analysis are shown in Figure 3. The values obtained by the sum of EAA, expressed as mg of amino acids on g of protein, were compared with the recommended intake for children (3–18 years old) and adults, which was calculated by the FAO (indicated as reference lines in the graph) (32).

The results highlighted that all fortified breads fully satisfy the needs reported by the FAO for adults and children (32). Going into more detail, it is possible to observe a statistical increase in the sum of EAA in samples A and B compared with controls C A and C B, respectively. Through the fortification process, the greatest increase was observed for sample B, which was indeed characterized by an increase of approximately 12% in EAA compared with its control (C B). Regarding samples D and E, although the slightly lower content in EAA showed compared with the corresponding control (C D and C E, respectively), no significant differences were observed (ρ < 0.05).

Despite the general increase in essential amino acid content, a decrease was observed in sample C. For this reason, the Amino Acid Score (AAS) of the different samples was also evaluated in relation to the needs determined by the FAO for children (3–18 years old) and adults (32). Table 8 shows the AAS values calculated, specifically, for lysine (LYS) and sulfur amino acids (SAA, sum of cysteine and methionine).

**Table 8:** Enantiomeric purity (D%) determined on control breads (on the left) and fortified breads (on the right) expressed as mean ± st. dev.

Enantiomeric purity (D%)					
Control breads			Fortified breads		
C A	ALA	2.10 ± 0.11	A	ALA	nd
	ASP	2.58 ± 0.11		ASP	6.56 ± 0.76
	GLU	1.10 ± 0.05		GLU	3.08 ± 0.75
	LYS	nd		LYS	nd
	PHE	0.55 ± 0.04		PHE	1.91 ± 0.23
	**TOT**	**6.33 ± 0.31**		**TOT**	11.5 ± 0.24*
C B	ALA	2.15 ± 0.14	B	ALA	n d
	ASP	2.17 ± 0.05		ASP	3.85 ± 0.22
	GLU	1.23 ± 0.07		GLU	1.19 ± 0.24
	LYS	nd		LYS	nd
	PHE	1.53 ± 0.07		PHE	1.99 ± 0.09
	**TOT**	**7.08 ± 0.31**		**TOT**	9.71 ± 0.55*
C C	ALA	1.41 ± 0.06	C	ALA	nd
	ASP	3.59 ± 0.07		ASP	7.72 ± 0.03
	GLU	1.16 ± 0.03		GLU	2.72 ± 0.11
	LYS	nd		LYS	nd
	PHE	1.34 ± 0.06		PHE	1.68 ± 0.26
	**TOT**	**7.50 ± 0.22**		**TOT**	12.1 ± 0.34*
C D	ALA	2.12 ± 0.03	D	ALA	nd
	ASP	3.23 ± 0.14		ASP	6.47 ± 0.44
	GLU	1.28 ± 0.06		GLU	2.24 ± 0.16
	LYS	nd		LYS	nd
	PHE	1.51 ± 0.07		PHE	1.95 ± 0.50
	**TOT**	**8.15 ± 0.31**		**TOT**	10.7 ± 1.11*
C E	ALA	2.65 ± 0.08	E	ALA	nd
	ASP	3.35 ± 0.17		ASP	6.81 ± 0.13
	GLU	1.70 ± 0.02		GLU	2.58 ± 0.13
	LYS	nd		LYS	2.67 ± 0.44
	PHE	1.55 ± 0.04		PHE	nd
	**TOT**	**9.26 ± 0.31**		**TOT**	12.1 ± 0.19*

Samples coding: (A) Spelt bread with pea-protein and walnuts (19% pea concentrate) and its control C A; (B) Spelt bread with pea-protein and sunflower seeds inside (19% pea concentrate) and its control C B; (C) Multi bread with chickpea-protein without yeast with cereal flakes and seeds (19% chickpea concentrate) and its control C C; (D) Three-grain bread (rye, wheat, oat) with chickpea-protein (19% chickpea concentrate) and its control C D; (E) Spelt-barley-bread with pea- and chickpea- protein with pumpkin and sesame seeds (16% pea and chickpea concentrate in 50:50 ratio) and its control C E. Asterisks in the table refer to the significant differences (ρ < 0.05) between the total D% of each sample and its control performed with pair *T*-test.

Looking at the results, almost all the amino acids analyzed showed values greater than or equal to 1, except for lysine, which remains the limiting amino acid even in fortified samples. However, moving from controls to fortified breads, an increase in the AAS of lysine of 68.2% for the needs of children and 67.9% in adults was observed, significantly improving the nutritional profile of the products under analysis.

### 3.6 Protein integrity

The protein integrity was evaluated by determining the degree of hydrolysis (DH%) and the enantiomeric purity (D %). The results for which concerns the DH% are shown in Figure 2, while the D % of the samples is shown in Table 3.

From the data obtained, it can be observed that regarding control and fortified breads, the DH% of the proteins, used as an indication of their possible degradation, was lower than 3%. This is an indication of the high integrity of the protein fraction present in the matrices under analysis. Generally, all the control breads showed a statistically greater (ρ < 0.05) DH% than fortified breads.

As can be observed from data shown in Table 3, the D% was observed to be statistically higher (ρ < 0.05) in all formulations for fortified breads. Generally, all control samples showed the presence of alanine D-enantiomers, which were not determined in the fortified samples. The presence of D-lysine was detected only in sample E, while the D-aspartic acid was the enantiomer showing the greatest values.

### 3.7 Protein digestibility

The results deriving from the application of the *in vitro* harmonized standard gastro-intestinal INFOGEST procedure were estimated due to the determination of the DH% after digestion. Data are shown in Figure 4 and are expressed as DH% of digestates at the net of the digestion blanks, therefore indicating the DH% due to the action of the digestive enzymes.

The DH% values are wide between samples, ranging from 20 up to 55%. The average DH% for control breads was approximately 42.6%, showing generally a better digestibility than fortified samples. Indeed, when comparing control breads with fortified breads, a decrease in the degree of hydrolysis of some samples can be noted. In particular, the DH% reported for B and E shows a notable decrease (ρ < 0.05) compared with the control bread. The most important change is found in the C E-digested sample within which a DH% of 55.3 ± 3.19% is detected which, following the fortification process, reaches a value equal to 34.92 ± 4.71%, confirming the lower protein digestibility of this formulation.

No significant difference (ρ < 0.05) was observed in the DH% reported for samples A, C, and D compared with the relative controls. In particular, sample C appears to be the one with the highest degree of hydrolysis comparable with the control (48.8 ± 1.1% versus 51.7 ± 2.3%, respectively).

## 4 Discussion

The data observed from the proximate composition are in agreement with what was observed in the literature. Xing et al. (35) observed that following the fortification process – in wheat bread fortified using dry fractionated chickpea protein-enriched fractions –, there is an increase in the total protein content of 38.5%, a figure comparable with that obtained in this work (equal to 39.5% on average). The decrease in the total carbohydrates in the fortified breads compared with the control ones can be attributed to the matrices. In particular, the partial replacement of flour (a matrix rich in carbohydrates) with protein concentrates (with a low content in carbohydrates) clearly modifies the chemical composition of bread. Indeed, carbohydrates are the major constituents of cereals, with starch making up 60% of the dry weight (36). Nevertheless, the total carbohydrate values observed in our samples are in agreement with what was reported by Plustea et al. (37), who evaluated the composition of breads fortified with lupine flour. In this study, in fact, the amount of carbohydrates found in the fortified breads was equal to 51.4 g/100 g, which was slightly lower than our data but completely comparable considering the different nature of the fortifying agent used.

The value of total lipids reported for the bread analyzed is quite higher than what is reported in the literature by Plustea et al. (37). Samples, and in particular sample C, showed a higher lipid content. This difference can be attributed to the presence of different types of oilseeds within these breads. Indeed, oilseeds are composed of at least 15% of fats (38). Being sample C the prototype with the highest content in oilseeds, the high concentration of fat in this product was an expected result. On the contrary, the formulation studied by Plustea et al. did not contain oilseeds.

The ash content detected within the samples does not appear to be influenced by the fortification process, and it does not vary when comparing control breads with the fortified ones. This highlights that the contribution of ash to the samples is not due to the protein concentrate. In fact, both control and fortified breads are composed of numerous seeds, which provide various mineral substances to the product (39). In this case, however, the data disagree with what was observed by Xing et al. (35) where the ash content detected was 2–3 g/100 g, slightly lower than the 3.9 g/100 g average observed in our study. However, it is necessary to note that the breads used in the aforementioned study are not characterized by the presence of seeds as in the case of the samples under examination but are wheat breads enriched with protein extracts.

The fortification process does not only potentially bring modifications to the composition of nutrients of products but can also affect the protein quality and integrity. For this reason, an in-depth study of the protein fraction of the samples under examination was considered essential. This characterization was conducted starting from the analysis of the protein profile (electrophoretic analysis in reducing conditions), the amino acid profile, and the integrity of the protein fraction.

In particular, the identification of the protein fraction highlighted the presence of pea protein concentrate of mostly vicilin, albumin, and legumin, the prevalent proteins in this matrix (17, 40, 41). In samples fortified with pea protein concentrates, it is possible to identify the presence of these proteins by comparison, but the bands appear less intense. This is certainly due to the composition characterized by ingredients which, in themselves, provide proteins to the system. On the contrary, the protein classes identified for the chickpea concentrate are clearly visible in the fortified products and belong to the class of vicilin-like and legumin-like proteins. These proteins are important storage proteins that are also characteristic of Leguminosae (42).

The differences observed in the total amino acid content are consistent with the trend observed in the proximate composition. Despite comparing the data observed for the protein content between the determination with the Kjeldahl method and the estimation with the sum of EAA, a great difference in the value was observed. From what has been highlighted, it can certainly be concluded that the value of the total protein content, which is determined through the Kjeldahl method, overestimates the real protein content of the different samples. In fact, the generic conversion factor of 6.25 appears to be too high in the specific case of these samples. For this reason, the data reported by the sum of total amino acids appears to be more reliable.

In addition to the values of total amino acids, the profile of the latter reported a higher content in almost all amino acids, which concerns fortified samples. This increment due to the fortification was observed also in the sum of EAA. Indeed, all samples fulfilled the requirements set by FAO for children (3–18 years old) and adults (32). In particular, samples fortified with pea protein concentrate (A and B) showed a higher increase in EAA than samples added with chickpea protein concentrate (C and D) or the mixture of both (E). The values of essential amino acids observed for the samples are in accordance with what was reported in the literature. In particular, Alu’datt et al. (43) evaluated the fortification of wheat breads with protein isolates produced from barley. In this study, the average value estimated for EAA increased by approximately 10.7%, which was comparable to the increment observed in the present study.

Despite the general trend observed in EAA for the samples, sample C showed a decreased content in EAA than its control bread (C C). In the study, therefore, the AAS was also evaluated. The AAS, indeed, gave an indication of the fulfillment of requirements for each amino acid. In particular, the results were reported for LYS and SAA, being generally limiting amino acids, respectively, in cereals and legumes (17, 36). The improvement in the profile observed is important especially in the case of sample C. Indeed, even if the sum of EAA resulted lower than the control bread, it can be noted that, due to fortification, there is an improvement in the proportion between the various amino acids, consequently improving the amino acid profile of the bread. In all cases, the increase in LYS score is accompanied by a corresponding decrease in the SAA value, which are limiting in legumes. This decrease does not, however, lead to a worsening of the nutritional profile of the breads, being the values still above 1.

The data presented so far are in accordance with what was reported by Guardado-Félix et al. (44). In this study, the partial replacement of wheat flour with chickpea flour in the formulation of fortified breads was evaluated. In addition, in this case, both in the controls and in the fortified samples analyzed, LYS was the limiting amino acid (AAS equal to approximately 0.5 in the controls), but, in general, the fortification process improved the nutritional profile of the breads, increasing the score up to approximately 66.2%, completely comparable to 68%, on average, observed in our study.

Analyzing what emerged from the evaluation of the DH% and the D% of the samples, the low values recorded indicated a high degree of purity and integrity of the protein fraction. In particular, the values observed for the DH% allow to state that the processes of protein extraction, fortification, flour milling, or bread baking did not significantly affect the protein integrity. The significant differences (ρ < 0.05) observed between controls and fortified breads can be ascribed to a greater DH% of the flour compared with the protein concentrates. Indeed, the latter, as mentioned previously, was subjected to DAE, a technique characterized by rather low degrees of protein hydrolysis. The protein fraction of the flour, on the other hand, has lower integrity due, among others, to the milling process, which has a greater impact in this sense (45). Consequently, by combining these two ingredients as foreseen by the fortification process, there is a decrease in the DH%.

For which concerns the D%, although this analysis is performed on all the amino acids present in the protein fraction of the samples, those detected were only the most sensitive to treatments (presented in Table 3). Following extensive treatments (such as high temperatures, ultrasounds, and extreme pH), the amino acids that are more susceptible to modifications are, indeed, alanine, aspartic acid, glutamic acid, lysine, and phenylalanine (46, 47). The higher values, in both control and fortified breads, were detected for D-aspartic acid, which were known to be very susceptible to heat treatments (48). From the data observed, a higher integrity of control breads was noted compared with fortified samples. The D% is related to all the treatments that the protein fraction underwent during the manufacturing. Therefore, these higher values observed in fortified breads can be ascribed to all the stress that the proteins have undergone from harvesting to extraction.

Finally, the digestibility of proteins was analyzed to have an indication of how and how much the process of fortification can affect it. To the aim, samples underwent the static *in vitro* INFOGEST gastro-intestinal digestion procedure, which is described in the methodology section (Section 2.8). The information on the protein digestibility was analyzed and expressed as DH% after digestion, net of the intrinsic DH% of samples prior to digestion. The results, as shown in Figure 4, showed a general lower, even if in some cases, statistically comparable digestibility of fortified breads than control formulations. Sample C was the sole fortified bread showing a comparable DH% of proteins compared with the control bread (C C). Regarding this factor, indeed, the fortification process seems to have worsened protein accessibility, affecting protein digestibility. What was observed in this study is in contrast to the literature. In particular, Sousa et al. (49) determined the digestibility of different protein isolates such as cereals and legumes with the INFOGEST method. In their study, the DH% of digestates reported for pea proteins was 40% higher than the DH% of cereals. These differences can be attributed to the matrix effect due to the final products, which is the result of the combination of different ingredients. In particular, the significant presence of complex carbohydrates (fiber) and lipids, interacting with proteins, could cause a slowdown in protein digestibility (50). Indeed, sample B, that showed the higher increase in total carbohydrate content, showed also a much lower digestibility than its control samples (C B). Nevertheless, sample E, made with the combination of pea and chickpea protein concentrates, showed lower DH% values compared with the control sample (C E). Interestingly, this is also the sample that showed the highest increase in protein content after fortification. Therefore, the lower digestibility could also be due to the presence of anti-nutritional factors provided by legume protein concentrates (18, 19), which could possibly have a higher impact on digestibility in the samples with higher proteins. Indeed, the presence of anti-nutritional factors as enzyme inhibitors (i.e., trypsin inhibitors) leads to a significant decrease in the activity of digestive enzymes (such as trypsin, chymotrypsin, amylase, and lipase) and a consequent decrease in the digestibility of the food matrix (22).

## 5 Conclusion

The partial replacement of cereal flours with protein concentrates led, as expected, to an increase in protein content, in accordance with what was estimated during the formulation to achieve the claim of “high protein content.”

The high integrity of protein observed—DH% lower than 3% and D% lower than 10%—and the general rebalancing of the profile in EAA—such as the increased AAS determined for lysine—achieved with the fortification led to a product with not only a high protein content but also a high-quality protein.

However, a high variability was observed in the protein digestibility attributable to the different composition of the samples. Beyond that, the lower digestibility observed in the fortified samples compared with the control breads suggests a possible residual presence of anti-nutritional factors in the samples due to the mild treatments applied to produce the protein concentrates.

We can conclude that the fortification led to a clear improvement in the nutritional profile of the bread prototypes. Regarding the digestibility of the protein fraction, sample C (fortified with chickpea proteins) is certainly the one that showed the best results, having a comparable DH% with its control bread.

These results show how the fortification of breads can be useful for improving the quality of these foods, the consumption of which is certainly a valid strategy to also increase the introduction of proteins with high biological value contained in legumes, representing an excellent tool in the fight against malnutrition. Anyhow, this study also highlighted the need to study more thoroughly the possibility of developing different formulations with different combinations of cereal-based and legume-based ingredients.

The products developed in this study are interesting; thus there is a literature about the use of protein concentrates to fortify bread, but not much has been done regarding the use of by-products. In this optic, a focus on the life-cycle assessment (LCA) of these new prototypes is needed to asses if, coupled with the nutritional advantages brought by fortification, there is also a beneficial effect on the environment.

Furthermore, this study was developed on five prototypes that were not yet completely food-grade. The recipe(s) of the products that deemed suitable must therefore be further studied and characterized also from technological and sensorial point of view. There is also the need to evaluate the digestibility of the starch, with particular attention to the possibility of having, with the fortification with protein concentrates, a lowering of the glycaemic index of breads. Finally, before they can be marketed, the products will require in-depth studies on stability and shelf life.

## Data availability statement

The original contributions presented in the study are included in the article/Supplementary material, further inquiries can be directed to the corresponding author.

## Author contributions

SC: Investigation, Methodology, Writing – original draft. J-IP: Investigation, Methodology, Writing – review & editing. RS: Resources, Writing – review & editing. CZ: Writing – review & editing. TT: Resources, Writing – review & editing.
