# Supplementary material for: Formulation of new sourdough bread prototypes fortified with non-compliant chickpea and pea residues
Source: Front Nutr. 2024 Jun 6;11:1351443. doi: 10.3389/fnut.2024.1351443 (PMC11200124; doi:10.3389/fnut.2024.1351443)
Supplement: Supplementary file 1 [file Image_1.pdf]

Supplementary material

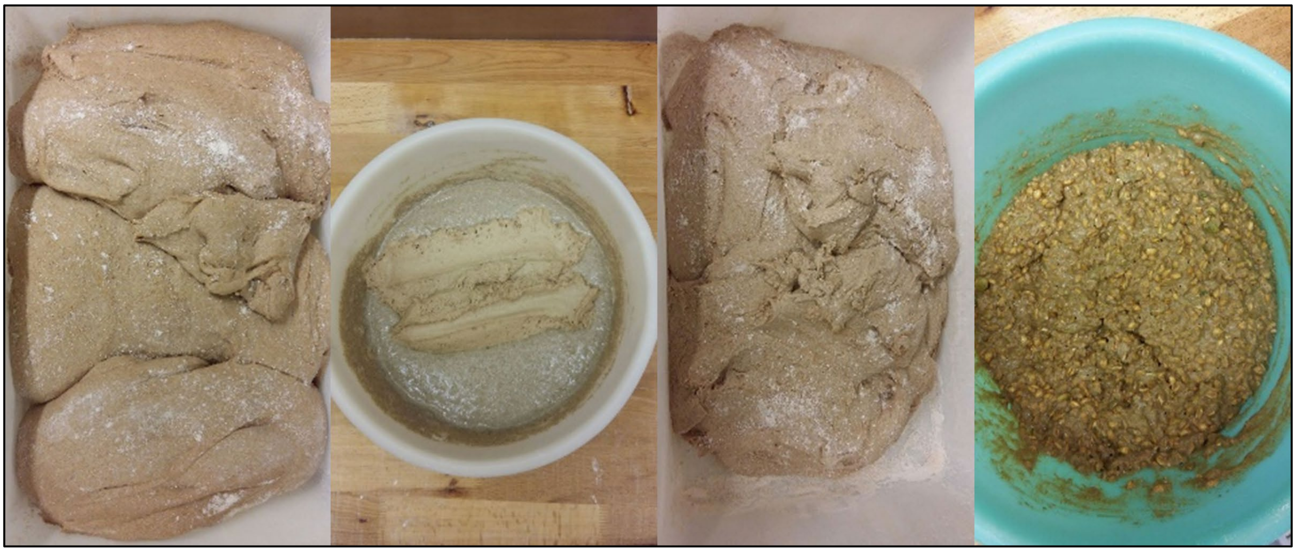

Figure S.1 *Sourdoughs added in the formulations.*

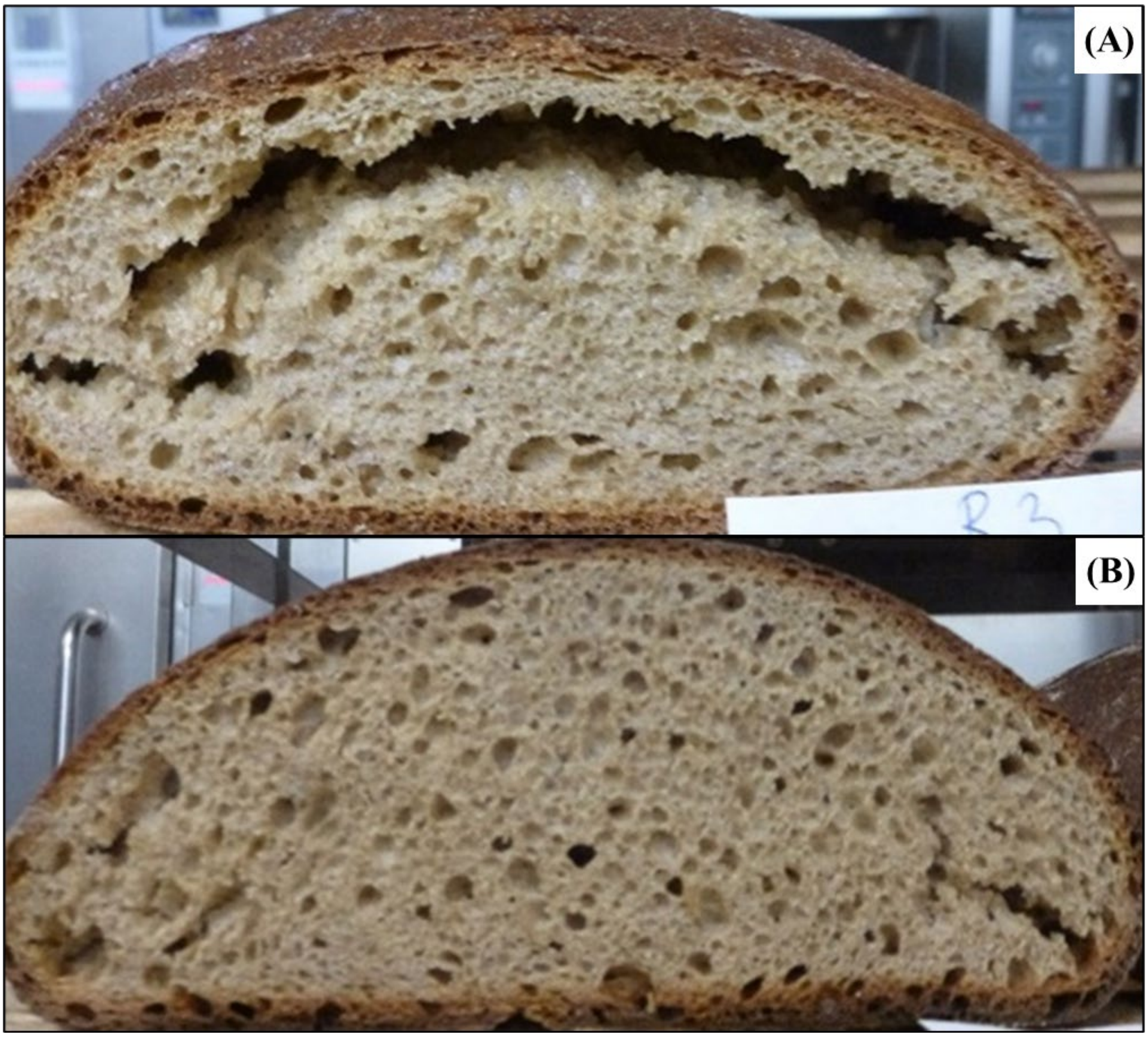

Figure S.2 *Comparison of bread loafs: standard baking test (panel A) and baking test with higher sourdough concentration (panel B).*

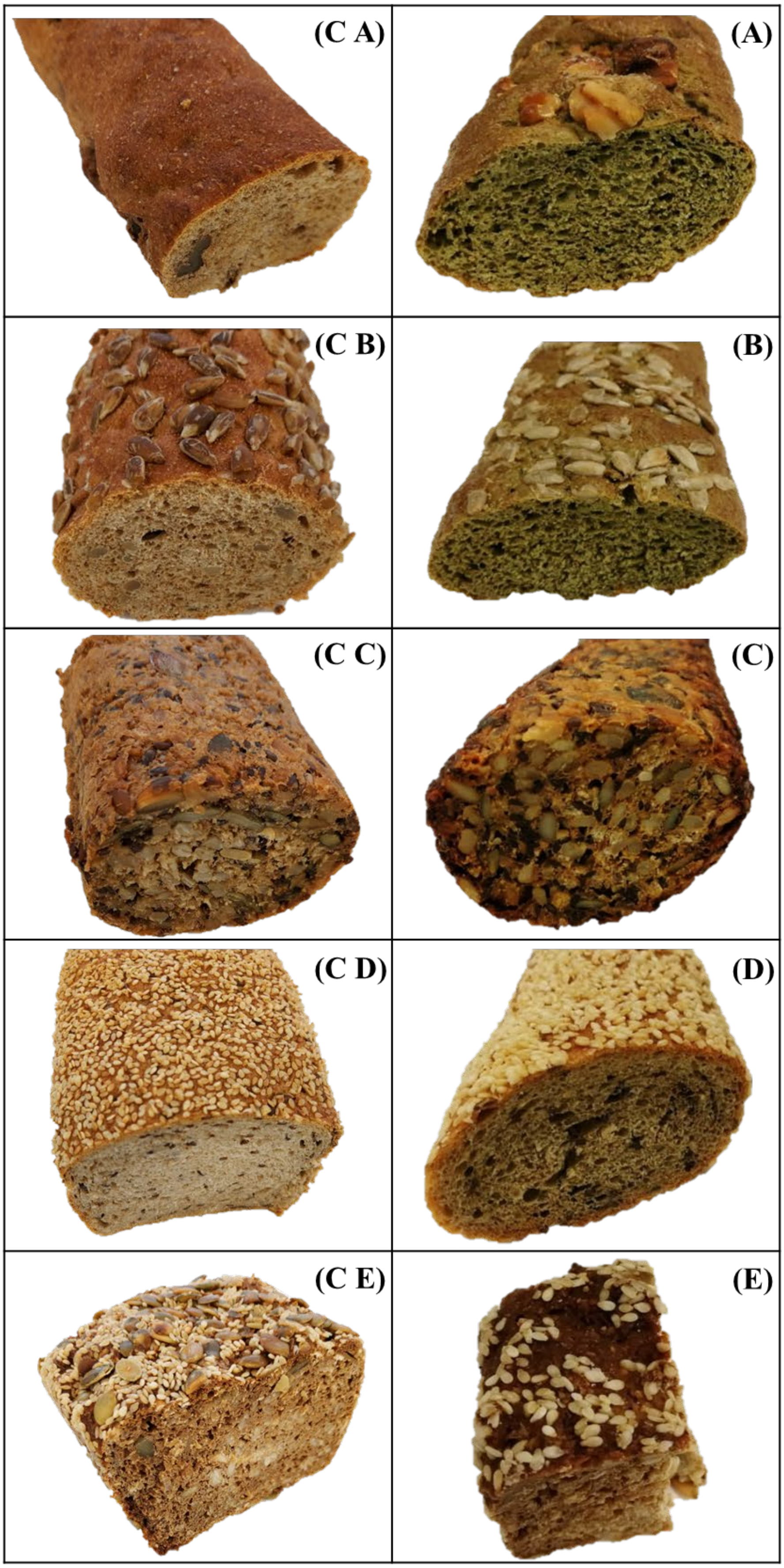

Figure S.3 *Control (on the left) and fortified (on the right) bread samples under investigation.*
